# Correction: Massively synthesizable nickel-doped 1T-MoS_2_ nanosheet catalyst as an efficient tri-functional catalyst

**DOI:** 10.1039/d3ra90058d

**Published:** 2023-07-04

**Authors:** Yusong Choi, Tae-Young Ahn, Ji-Youn Kim, Eun Hye Lee, Hye-Ryeon Yu

**Affiliations:** a Defense Materials and Energy Development Center, Agency for Defense Development Yuseong P.O. Box 35 Daejeon 34060 Korea richpine87@gmail.com; b Department of Defense System Engineering, University of Science and Technology Daejeon 34113 Korea

## Abstract

Correction for ‘Massively synthesizable nickel-doped 1T-MoS_2_ nanosheet catalyst as an efficient tri-functional catalyst’ by Yusong Choi *et al.*, *RSC Adv.*, 2023, **13**, 18122–18127, https://doi.org/10.1039/D3RA03016D.

The authors regret that the affiliations for Ji-Youn Kim and Hye-Ryeon Yu were incorrectly shown in the original manuscript. The corrected list of affiliations is as shown here.

The Royal Society of Chemistry apologises for these errors and any consequent inconvenience to authors and readers.

## Supplementary Material

